# Associations between normal weight central obesity and cardiovascular disease risk factors in Japanese middle-aged adults: a cross-sectional study

**DOI:** 10.1186/s41043-019-0201-5

**Published:** 2019-12-18

**Authors:** Takako Shirasawa, Hirotaka Ochiai, Takahiko Yoshimoto, Satsue Nagahama, Mariko Kobayashi, Iichiro Ohtsu, Yuma Sunaga, Akatsuki Kokaze

**Affiliations:** 1 Department of Hygiene, Public Health and Preventive Medicine, Showa University School of Medicine, 1-5-8 Hatanodai, Shinagawa-ku, Tokyo, 142-8555 Japan; 2 All Japan Labor Welfare Foundation, 6-16-11 Hatanodai, Shinagawa-ku, Tokyo, 142-0064 Japan

**Keywords:** Normal weight central obesity, Body mass index, Waist-to-height ratio, Cardiovascular disease, Hypertension, Dyslipidemia, Diabetes

## Abstract

**Background:**

Several studies have shown that normal weight central obesity (NWCO) is associated with cardiovascular disease (CVD) risk factors. However, studies conducted in the Japanese population have been very limited. Thus, the relationships between normal weight central obesity, classified using body mass index (BMI), the waist-to-height ratio (WHtR), and CVD risk factors in middle-aged Japanese adults were investigated.

**Methods:**

The participants were Japanese adults aged 40–64 years who had undergone periodic health examinations in Japan during the period from April 2013 to March 2014. The participants were categorized into the following four groups: normal weight (BMI 18.5–24.9 kg/m^2^) and no central obesity (WHtR < 0.5) (NW); normal weight and central obesity (WHtR ≥ 0.5) (NWCO); obesity (BMI ≥ 25 kg/m^2^) and no central obesity (OB); and obesity and central obesity (OBCO). Hypertension was defined as systolic blood pressure ≥ 140 mmHg, diastolic blood pressure ≥ 90 mmHg, or taking medication for hypertension. Dyslipidemia was defined as LDL-C ≥ 140 mg/dl, HDL-C < 40 mg/dl, triglyceride ≥ 150 mg/dl, or taking medication for dyslipidemia. Diabetes was defined as fasting plasma glucose ≥ 126 mg/dl, random plasma glucose ≥ 200 mg/dl, HbA1c ≥ 6.5%, or receiving medical treatment for diabetes mellitus. A logistic regression model was used to calculate the odds ratios (ORs) and 95% confidence intervals (CIs) for hypertension, dyslipidemia, and diabetes.

**Results:**

A total of 117,163 participants (82,487 men and 34,676 women) were analyzed. The prevalence of NWCO was 15.6% in men and 30.2% in women. With reference to NW, the ORs for hypertension (adjusted OR 1.22, 95% CI 1.17–1.27 in men, 1.23, 1.16–1.31 in women), dyslipidemia (1.81, 1.74–1.89 in men, 1.60, 1.52–1.69 in women), and diabetes (1.35, 1.25–1.46 in men, 1.60, 1.35–1.90 in women) were significantly higher in NWCO.

**Conclusions:**

Normal weight with central obesity was associated with CVD risk factors, such as hypertension, dyslipidemia, and diabetes, compared with normal weight without central obesity, regardless of sex. It is important to focus on normal weight with central obesity for the prevention of CVD in Japanese middle-aged adults.

## Background

In the general population, obesity is consistently and strongly related to higher risks of cardiovascular disease (CVD) incidence and death [1]. By contrast, a recent study showed that abdominal obesity is associated with insulin resistance and higher risks of metabolic syndrome and CVD, whereas general obesity is not [2]. Moreover, “normal weight central obesity,” defined by considering general obesity and central obesity, has been shown to be associated with CVD risk factors and increased mortality [3]. Thus, it can be effective to consider both general obesity and central obesity for prevention of CVD.

General obesity is most commonly assessed using the body mass index (BMI) [4]. Although the BMI is strongly correlated with gold standard body fat measures, it cannot distinguish between lean and fat mass and provides no indication of body fat distribution [4]. By contrast, abdominal obesity is assessed using indicators such as waist circumference (WC), waist-to-hip ratio (WHR), and waist-to-height ratio (WHtR) [5]. A previous systematic review demonstrated that, as indices of abdominal obesity, the WHtR was a better predictor than BMI and WC for diabetes, dyslipidemia, hypertension, and CVD in both sexes in populations of various nationalities [6]. The WHtR may be a simpler and better predictor of early health risks [7–9]. Thus, it is effective to use BMI and the WHtR for defining general obesity and central obesity.

Several studies reported that normal weight central obesity defined by BMI and WHR [10–12], body fat percentage [13, 14], and WC [15–17] were associated with CVD risk factors. By contrast, there were a few studies regarding the association of normal weight central obesity defined by BMI and WHtR with CVD risk factors [18–20]. Moreover, to the best of our knowledge, studies conducted in the Japanese population have been very limited.

Accordingly, the aim of the present study was to investigate the prevalence of normal weight central obesity classified using BMI and the WHtR and to examine the relationships between normal weight with central obesity and CVD risk factors, especially hypertension, dyslipidemia, and diabetes, in middle-aged Japanese adults. We hypothesized that, in Japanese men and women, those with normal weight and central obesity have a higher risk of CVD risk factors compared with those with normal weight and no central obesity or obesity with no central obesity.

## Methods

### Subjects and setting

The subjects of this study were Japanese men and women aged 40–64 years who had undergone periodic health examinations provided by the All Japan Labor Welfare Foundation (Tokyo), a health service center in Japan, during the period from April 2013 to March 2014. Written, informed consent was obtained from the subjects. The study protocol was approved by the Medical Ethics Committee of Showa University School of Medicine (Approval No. 2132) and the Ethics Committee of the All Japan Labor Welfare Foundation (Approval No. 3-1-0004).

### Variables and their measurement

The following information was obtained from each subject using a self-administered questionnaire, which was recommended for specific health examination by the Japanese government (Ministry of Health, Labour and Welfare) [21]: age, sex, smoking status (current smoker, ex-smoker, non-smoker), alcohol intake (daily, sometimes, none), and physical activity equal to walking at least 60 min per day (yes, no).

Height and weight were measured in increments of 0.1 cm and 0.1 kg, respectively, by trained staff. BMI was calculated as the weight (kg) divided by the squared height (m^2^). WC was measured to the nearest 0.1 cm at the umbilical level in a standing position [22]. The WHtR was calculated as WC divided by height. Blood pressure in the sitting position was measured using an automated machine (HEM-907, Omron, Kyoto, Japan).

Venous blood samples were drawn from the study subjects to measure serum levels of high-density lipoprotein cholesterol (HDL-C), low-density lipoprotein cholesterol (LDL-C), triglycerides, blood glucose, and hemoglobin A1c (HbA1c). The samples were stored in a cooler at 4 °C for transportation to at an external laboratory (SRL, Tokyo, Japan) and measured within 24 h of being drawn. HDL-C and LDL-C were determined by a direct method, while the triglyceride level was measured by an enzyme method (AU5400, BECKMAN COULTER, Brea, CA, USA). The blood glucose level was obtained by the hexokinase method (AU5400, BECKMAN COULTER), while HbA1c was measured by a latex agglutination method (JCA-BM9130, JEOL, Tokyo, Japan).

Hypertension was defined as systolic blood pressure ≥ 140 mmHg, diastolic blood pressure ≥ 90 mmHg, or taking medication for hypertension [23]. Dyslipidemia was defined as LDL-C ≥ 140 mg/dl, HDL-C < 40 mg/dl, triglycerides ≥ 150 mg/dl, or taking medication for dyslipidemia [24]. Diabetes was defined as fasting plasma glucose (≥ 8 h after the last caloric intake) ≥ 126 mg/dl, random plasma glucose ≥ 200 mg/dl, HbA1c (National Glycohemoglobin Standardization Program) ≥ 6.5%, or receiving medical treatment for diabetes mellitus [25, 26].

### Definition of normal weight central obesity

BMI was categorized into three groups: < 18.5 (underweight), 18.5–24.9 (normal weight), and ≥ 25 kg/m^2^ (obesity) [27]. WHtR was dichotomized as follows: < 0.5 (no central obesity) and ≥ 0.5 (central obesity) [28, 29]. Moreover, according to previous studies [19, 20], the subjects were categorized into the following four groups: normal weight and no central obesity (NW); normal weight and central obesity (NWCO); obesity and no central obesity (OB); and obesity and central obesity (OBCO). In the present study, underweight subjects (BMI < 18.5 kg/m^2^) were excluded from the analysis.

### Statistical analysis

The Kruskal-Wallis test or chi-squared test was used to compare the characteristics among the four groups (NW, NWCO, OB, and OBCO) for each sex. In the analysis stratified by sex, a logistic regression model was used to calculate the odds ratios (ORs) and the 95% confidence intervals (CIs) for hypertension, dyslipidemia, and diabetes. In the model, age, weight, smoking status, alcohol intake, and physical activity were included to control for potential confounding factors [19].

In this study, a *P* value of less than 0.05 was considered significant. All data were analyzed using JMP version 13.0 (SAS Institute Japan Co., Ltd., Tokyo, Japan).

## Results

Of the 310,577 subjects, 310,498 participated in this study. From among these participants, 185,430 participants with missing data and 7905 participants who were underweight (BMI < 18.5 kg/m^2^) were excluded. Thus, a total of 117,163 participants (82,487 men and 34,676 women) were analyzed.

The median WC was higher in men than in women, whereas the median WHtR was higher in women than in men (Table 1). The proportions of NW, NWCO, OB, and OBCO were 50.8%, 19.9%, 1.6%, and 27.7%. The proportions were 52.2%, 15.6%, 2.1%, and 30.1% in men and 47.6%, 30.2%, 0.3%, and 21.8% in women, respectively.

**Table 1:** Characteristics of participants by sex

	Men (*N* = 82,487)		Women (*N* = 34,676)	
Age (y)	50.0	(44.0, 56.0)	50.0	(45.0, 57.0)
Height (cm)	169.8	(165.7, 173.8)	157.2	(153.5, 161.3)
Weight (kg)	67.9	(61.6, 75.1)	55.0	(50.2, 61.7)
BMI (kg/m^2^)	23.5	(21.6, 25.7)	22.1	(20.3, 24.6)
WC (cm)	84.0	(78.5, 90.0)	79.0	(74.0, 85.5)
WHtR	0.495	(0.463, 0.530)	0.503	(0.469, 0.544)
NW	43,055	(52.2)	16,513	(47.6)
NWCO	12,877	(15.6)	10,483	(30.2)
OB	1714	(2.1)	117	(0.3)
OBCO	24,841	(30.1)	7563	(21.8)
Hypertension	30,739	(37.3)	10,282	(29.7)
Dyslipidemia	44,848	(54.4)	14,720	(42.5)
Diabetes	7457	(9.0)	1443	(4.2)
Smoking status				
Current smoker	35,853	(43.5)	6164	(17.8)
Ex-smoker	16,172	(19.6)	2428	(7.0)
Non-smoker	30,462	(36.9)	26,084	(75.2)
Alcohol intake				
Daily	34,930	(42.4)	5408	(15.6)
Sometimes	25,446	(30.9)	10,382	(29.9)
None	22,111	(26.8)	18,886	(54.5)
Physical activity				
Yes	28,108	(34.1)	10,567	(30.5)
No	54,379	(65.9)	24,109	(69.5)

Data are expressed as medians (25th percentile, 75th percentile) or *n* (%)

*BMI*, body mass index; *WC*, waist circumference; *WHtR*, waist-to-height ratio; *NW*, normal weight no central obesity; *NWCO*, normal weight central obesity; *OB*, obesity no central obesity; *OBCO*, obesity central obesity

The characteristics of the four groups classified by BMI and WHtR (NW, NWCO, OB, and OBCO) are shown in Table 2 for men and Table 3 for women. The prevalences of hypertension, dyslipidemia, and diabetes in OBCO were 48.9%, 68.9%, and 16.2% in men and 43.8%, 57.6%, and 11.0% in women, respectively. The prevalences of hypertension, dyslipidemia, and diabetes were higher in OBCO than in NW, NWCO, and OB, regardless of sex. The prevalences in NWCO were 39.6%, 60.1%, and 9.6% in men and 31.0%, 48.4%, and 3.6% in women, respectively. The participants with NWCO had higher prevalences of hypertension, dyslipidemia, and diabetes than those with NW, regardless of sex.

**Table 2:** Characteristics of the four groups classified by BMI and WHtR in men (*N* = 82,487)

	NW (*n* = 43,055)		NWCO (*n* = 12,877)		OB (*n* = 1714)		OBCO (*n* = 24,841)		*p* value*
Age (y)	49.0	(44.0, 55.0)	54.0	(48.0, 60.0)	46.0	(43.0, 51.0)	50.0	(44.0, 56.0)	< 0.001
Height (cm)	170.4	(166.4, 174.4)	167.5	(163.4, 171.6)	172.5	(169.0, 176.2)	169.7	(165.6, 173.6)	< 0.001
Weight (kg)	63.1	(58.5, 67.8)	66.9	(62.8, 70.7)	76.2	(73.0, 80.0)	78.8	(73.7, 85.5)	< 0.001
BMI (kg/m^2^)	21.7	(20.5, 22.9)	23.9	(23.1, 24.4)	25.5	(25.2, 25.9)	27.1	(25.9, 29.0)	< 0.001
WC (cm)	79.0	(75.0, 82.0)	86.5	(84.0, 89.0)	84.2	(82.0, 86.4)	93.2	(89.5, 98.0)	< 0.001
WHtR	0.465	(0.443, 0.482)	0.514	(0.506, 0.526)	0.492	(0.484, 0.496)	0.549	(0.528, 0.577)	< 0.001
Hypertension	12,868	(29.9)	5098	(39.6)	626	(36.5)	12,147	(48.9)	< 0.001
Dyslipidemia	18,970	(44.1)	7743	(60.1)	1017	(59.3)	17,118	(68.9)	< 0.001
Diabetes	2112	(4.9)	1231	(9.6)	93	(5.4)	4021	(16.2)	< 0.001
Smoking status									
Current smoker	19,622	(45.6)	5254	(40.8)	706	(41.2)	10,271	(41.4)	< 0.001
Ex-smoker	7660	(17.8)	2809	(21.8)	347	(20.3)	5356	(21.6)	
Non-smoker	15,773	(36.6)	4814	(37.4)	661	(38.6)	9214	(37.1)	
Alcohol intake									
Daily	19,304	(44.8)	6117	(47.5)	617	(36.0)	8892	(35.8)	< 0.001
Sometimes	12,906	(30.0)	3486	(27.1)	629	(36.7)	8425	(33.9)	
None	10,845	(25.2)	3274	(25.4)	468	(27.3)	7524	(30.3)	
Physical activity									
Yes	15,711	(36.5)	4048	(31.4)	705	(41.1)	7644	(30.8)	<0.001
No	27,344	(63.5)	8829	(68.6)	1009	(58.9)	17,197	(69.2)	

Data are expressed as medians (25th percentile, 75th percentile) or *n* (%)

*Kruskal-Wallis test or chi-squared test

*NW*, normal weight no central obesity; *NWCO*, normal weight central obesity; *OB*, obesity no central obesity; *OBCO*, obesity central obesity; *BMI*, body mass index; *WC*, waist circumference; *WHtR*, waist-to-height ratio

**Table 3:** Characteristics of the four groups classified by BMI and WHtR in women (*N* = 34,676)

	NW (*n* = 16,513)		NWCO (*n* = 10,483)		OB (*n* = 117)		OBCO (*n* = 7563)		*p* value
Age (y)	48.0	(44.0, 54.0)	54.0	(47.0, 59.0)	46.0	(43.0, 50.0)	51.0	(45.0, 57.0)	< 0.001
Height (cm)	158.5	(154.7, 162.3)	155.7	(152.2, 159.5)	164.2	(158.3, 170.2)	156.7	(152.7, 160.9)	< 0.001
Weight (kg)	51.4	(48.1, 55.2)	55.2	(51.7, 59.0)	69.0	(65.0, 73.9)	67.9	(63.0, 74.7)	< 0.001
BMI (kg/m^2^)	20.4	(19.5, 21.5)	22.8	(21.7, 23.8)	25.4	(25.1, 25.9)	27.2	(25.9, 29.5)	< 0.001
WC (cm)	74.0	(70.5, 76.5)	82.2	(79.9, 85.0)	80.0	(78.0, 83.5)	91.2	(87.0, 97.0)	< 0.001
WHtR	0.467	(0.447, 0.483)	0.524	(0.511, 0.543)	0.491	(0.484, 0.497)	0.581	(0.553, 0.617)	< 0.001
Hypertension	3678	(22.3)	3254	(31.0)	36	(30.8)	3314	(43.8)	< 0.001
Dyslipidemia	5235	(31.7)	5071	(48.4)	55	(47.0)	4359	(57.6)	< 0.001
Diabetes	229	(1.4)	380	(3.6)	5	(4.3)	829	(11.0)	< 0.001
Smoking status									
Current smoker	3149	(19.1)	1603	(15.3)	31	(26.5)	1381	(18.3)	< 0.001
Ex-smoker	1128	(6.8)	722	(6.9)	15	(12.8)	563	(7.4)	
Non-smoker	12,236	(74.1)	8158	(77.8)	71	(60.7)	5619	(74.3)	
Alcohol intake									
Daily	2921	(17.7)	1569	(15.0)	28	(23.9)	890	(11.8)	< 0.001
Sometimes	5093	(30.8)	3077	(29.4)	34	(29.1)	2178	(28.8)	
None	8499	(51.5)	5837	(55.7)	55	(47.0)	4495	(59.4)	
Physical activity									
Yes	5226	(31.7)	3197	(30.5)	30	(25.6)	2114	(28.0)	< 0.001
No	11,287	(68.4)	7286	(69.5)	87	(74.4)	5449	(72.1)	

Data are expressed as medians (25th percentile, 75th percentile) or *n* (%)

*Kruskal-Wallis test or chi-squared test

*NW*, normal weight no central obesity; *NWCO*, normal weight central obesity; *OB*, obesity no central obesity; *OBCO*, obesity central obesity; *BMI*, body mass index; *WC*, waist circumference; *WHtR*, waist-to-height ratio

Next, logistic regression analysis was conducted to calculate the crude and adjusted ORs for hypertension, dyslipidemia, and diabetes and their 95% CIs in each sex (Table 4 for men and Table 5 for women). When compared with NW, the adjusted ORs for hypertension (adjusted OR 1.58, 95% CI 1.51–1.65 in men; 1.55, 1.43–1.69 in women), dyslipidemia (1.84, 1.76–1.93 in men; 1.85, 1.70–2.01 in women), and diabetes (1.83, 1.70–1.97 in men; 3.11, 2.57–3.77 in women) were significantly increased in OBCO, regardless of sex. With reference to NW, the ORs for hypertension (1.22, 1.17–1.27 in men; 1.23, 1.16–1.31 in women), dyslipidemia (1.81, 1.74–1.89 in men; 1.60, 1.52–1.69 in women), and diabetes (1.35, 1.25–1.46 in men; 1.60, 1.35–1.90 in women) were significantly increased in NWCO.

**Table 4:** Odds ratios and their 95% confidence intervals for hypertension, dyslipidemia, and diabetes in men (*N* = 82,487)

				Crude		Adjusted	
	Total	*n*	(%)	OR	95% CI	OR	95% CI
Hypertension							
NW	43,055	12,868	(29.9)	1		1	
NWCO	12,877	5098	(39.6)	1.54	1.48–1.60	1.22	1.17–1.27
OB	1714	626	(36.5)	1.35	1.22–1.49	1.15	1.04–1.28
OBCO	24,841	12,147	(48.9)	2.24	2.17–2.32	1.58	1.51–1.65
Dyslipidemia							
NW	43,055	18,970	(44.1)	1		1	
NWCO	12,877	7743	(60.1)	1.91	1.84–1.99	1.81	1.74–1.89
OB	1714	1017	(59.3)	1.85	1.68–2.04	1.33	1.20–1.47
OBCO	24,841	17,118	(68.9)	2.81	2.72–2.91	1.84	1.76–1.93
Diabetes							
NW	43,055	2112	(4.9)	1		1	
NWCO	12,877	1231	(9.6)	2.05	1.90–2.20	1.35	1.25–1.46
OB	1714	93	(5.4)	1.11	0.90–1.38	0.83	0.66–1.03
OBCO	24,841	4021	(16.2)	3.74	3.54–3.96	1.83	1.70–1.97

Adjusted for age, weight, smoking status, alcohol intake, and physical activity

*NW*, normal weight no central obesity; *NWCO*, normal weight central obesity; *OB*, obesity no central obesity; *OBCO*, obesity central obesity; *OR*, odds ratio; *95% CI*, 95% confidence interval

**Table 5:** Odds ratios and their 95% confidence intervals for hypertension, dyslipidemia, and diabetes in women (*N* = 34,676)

				Crude		Adjusted	
	Total	*n*	(%)	OR	95% CI	OR	95% CI
Hypertension							
NW	16,513	3678	(22.3)	1		1	
NWCO	10,483	3254	(31.0)	1.57	1.49–1.66	1.23	1.16–1.31
OB	117	36	(30.8)	1.55	1.05–2.30	0.98	0.66–1.47
OBCO	7563	3314	(43.8)	2.72	2.57–2.89	1.55	1.43–1.69
Dyslipidemia							
NW	16,513	5235	(31.7)	1		1	
NWCO	10,483	5071	(48.4)	2.02	1.92–2.12	1.60	1.52–1.69
OB	117	55	(47.0)	1.91	1.33–2.75	1.42	0.98–2.06
OBCO	7563	4359	(57.6)	2.93	2.77–3.10	1.85	1.70–2.01
Diabetes							
NW	16,513	229	(1.4)	1		1	
NWCO	10,483	380	(3.6)	2.67	2.27–3.16	1.60	1.35–1.90
OB	117	5	(4.3)	3.17	1.28–7.85	1.77	0.71–4.44
OBCO	7563	829	(11.0)	8.78	7.54–10.16	3.11	2.57–3.77

Adjusted for age, weight, smoking status, alcohol intake, and physical activity

*NW*, normal weight no central obesity; *NWCO*, normal weight central obesity; *OB*, obesity no central obesity; *OBCO*, obesity central obesity; *OR*, odds ratio; *95% CI*, 95% confidence interval

## Discussion

In our study, the prevalence of NWCO defined using a combination of BMI and WHtR was 19.9%. The prevalence was higher than the reported NWCO prevalence in Thailand (15.4%) [20], whereas it was lower than that in South Africa (29.5%) [19]. One of the reasons could be the difference in the BMI cut-off level. The cut-off level in the Thai study was BMI < 25.0 kg/m^2^, and thus underweight subjects were included in the normal weight group. Moreover, in the South African study, the method for measurement of WC was different from that of the present study. WC in this study was measured at the umbilical level in a standing position [20], while it was measured at the level of the narrowest point between the lower costal border and the iliac crest in the South African study [19]. Therefore, the differences in the BMI cut-off level and the method of WC measurement might have affected the prevalence of NWCO.

In the present study, the prevalence of NWCO was higher in women than in men (30.2% vs. 15.6%). One possible explanation for the result might be that, in the present study, the proportion of normal weight was higher in women than in men (77.8% vs. 67.8%), and the proportion of central obesity was higher in women than in men (52.0% vs. 45.7%). However, the result that women had a higher prevalence of NWCO than men is not consistent with previous studies [18, 22]. Future studies will be needed to elucidate the sex differences in the prevalence of NWCO.

Normal weight with central obesity was associated with CVD risks such as hypertension, dyslipidemia, and diabetes in the present study; the ORs for hypertension, dyslipidemia, and diabetes were significantly increased in NWCO, as well as in OBCO, compared with NW, regardless of sex. These results were consistent with previous studies [18, 20, 30]. The present study suggested that using a combination of measures, including a measure of general obesity and a measure of central obesity, would be more appropriate in identifying CVD risk factors. Thus, the findings of the present study suggest that those who are NWCO need to be screened like those who are OBCO. Because these individuals with NWCO are considered normal weight, namely non-overweight/obesity, they do not usually receive the appropriate health education and prompt intervention to prevent the CVD risk factors. Moreover, a previous study reported that WHtR and BMI are independently associated with CVD risk [31]. Therefore, it is important to conduct screening for NWCO using a combination of BMI and WHtR [7–9] and intervene actively to prevent CVD risks such as hypertension, diabetes, and dyslipidemia.

To the best of our knowledge, this is the first study to investigate the prevalence of normal weight with central obesity and to examine the relationships between normal weight with central obesity classified using BMI and WHtR and CVD risk factors (especially hypertension, dyslipidemia, and diabetes) in middle-aged adults in Japan. A strength of the present study was the large sample size (over 110,000 participants), which contributed to a decrease in random error. Moreover, height, weight, and WC of study participants were measured by trained technicians, and these anthropometric variables were used to define obesity and central obesity. By contrast, some limitations of the present study should be noted. First, potential confounding factors that were not obtained in the present study might have affected the study’s findings. For instance, the information on dietary intake [32] and socioeconomic status [16], which has been reported to be associated with CVD risk factors, was not collected. Second, the study design was cross-sectional, which makes it difficult to examine causal relationships. Thus, further studies, including prospective studies, will be needed to establish causality.

## Conclusions

In conclusion, the present study showed that normal weight with central obesity was associated with CVD risk factors, such as hypertension, dyslipidemia, and diabetes, compared with normal weight without central obesity, regardless of sex. The present findings suggest that it is important to focus on normal weight with central obesity defined using a combination of BMI and WHtR to prevent CVD in Japanese middle-aged adults.

## Data Availability

The data used for this study are available on reasonable request and only after approval by the Ethics Committee of the All Japan Labor Welfare Foundation.

## Abbreviations

95% CI: 95% confidence interval
BMI: Body mass index
CVD: Cardiovascular disease
HbA1c: Hemoglobin A1c
HDL-C: High-density lipoprotein cholesterol
LDL-C: Low-density lipoprotein cholesterol
NW: Normal weight and no central obesity
NWCO: Normal weight and central obesity
OB: Obesity and no central obesity
OBCO: Obesity and central obesity
ORs: Odds ratios
WC: Waist circumference
WHR: Waist-to-hip ratio
WHtR: Waist-to-height ratio
